# Green synthesis of gold nanoparticles by thermophilic filamentous fungi

**DOI:** 10.1038/s41598-018-22112-3

**Published:** 2018-03-02

**Authors:** Zsófia Molnár, Viktória Bódai, George Szakacs, Balázs Erdélyi, Zsolt Fogarassy, György Sáfrán, Tamás Varga, Zoltán Kónya, Eszter Tóth-Szeles, Rózsa Szűcs, István Lagzi

**Affiliations:** 1 Fermentia Ltd, Budapest, Hungary; 2 Centre for Energy Research, Institute of Technical Physics and Materials Science, Budapest, Hungary; 3 Department of Applied and Environmental Chemistry, University of Szeged, Szeged, Hungary; 4 MTA-SZTE Reaction Kinetics and Surface Chemistry Research Group, University of Szeged, Szeged, Hungary; 5 Department of Physics, Budapest University of Technology and Economics, Budapest, Hungary; 6 MTA-BME Computer Driven Chemistry Research Group, Budapest University of Technology and Economics, H-1111 Szent Gellért tér 4 Budapest, Hungary; 7 MTA-BME Condensed Matter Research Group, Budapest University of Technology and Economics, Budapest, Hungary

## Abstract

Alternative methods, including green synthetic approaches for the preparation of various types of nanoparticles are important to maintain sustainable development. Extracellular or intracellular extracts of fungi are perfect candidates for the synthesis of metal nanoparticles due to the scalability and cost efficiency of fungal growth even on industrial scale. There are several methods and techniques that use fungi-originated fractions for synthesis of gold nanoparticles. However, there is less knowledge about the drawbacks and limitations of these techniques. Additionally, identification of components that play key roles in the synthesis is challenging. Here we show and compare the results of three different approaches for the synthesis of gold nanoparticles using either the extracellular fraction, the autolysate of the fungi or the intracellular fraction of 29 thermophilic fungi. We observed the formation of nanoparticles with different sizes (ranging between 6 nm and 40 nm) and size distributions (with standard deviations ranging between 30% and 70%) depending on the fungi strain and experimental conditions. We found by using ultracentrifugal filtration technique that the size of reducing agents is less than 3 kDa and the size of molecules that can efficiently stabilize nanoparticles is greater than 3 kDa.

## Introduction

Synthesis of nanoparticles (NPs) and their self-assembly into nanostructured materials are amongst the most studied topics in chemistry, physics and material science^1–6^. These particles and materials have unique optical, magnetic, electronic and chemical properties and have been used in various applications (e.g., solar cells, photovoltaic devices, heterogeneous catalysts, medicine)^7–10^. For examples, in chemical catalysis the catalytic activity of the NPs depends on the particle size and shape (this property is due to high surface area per unit volume and the nature of crystal planes exposed in catalysis)^11^. It is also well-known in biology and medicine that the cytotoxicity of gold nanoparticles (AuNPs) is size dependent^12^. Additionally, in optical properties of NPs, the plasmon absorption of AuNPs has size and shape dependence^13^.

The most frequently used chemical method for the synthesis of NPs is a wet synthetic method, in which reduction or co-precipitation process takes place for the generation of NPs^14^. The main drawbacks of this synthetic approach are the use of harsh chemical conditions and organic solvents and the production of toxic residues during the synthesis and functionalization of NPs. Therefore, in the last two decades alternative (green/biological) methods have been introduced and widely used in the production of various types of NPs^15–24^. These methods use microorganisms or extracts from various plants and intracellular or extracellular extracts of fungi or bacteria. Fungi are good candidates for large scale production of NPs because of the simplicity and the cost efficiency of growth on both the laboratory and the industrial scale.

Filamentous fungi can produce a wide variety of metal NPs such as gold^25–27^, silver^28,29^, iron oxide^30^ and even bimetallic nanoparticles^31^. Generally, fungi-mediated synthesis of NPs falls into two main categories: *in vivo* and *in vitro* methods. In the first case, the formation of NPs occurs intracellularly inside the living mycelia, and this phenomenon occurs because most transition metal ions are toxic to the organisms, processing them to a less-toxic form is advantageous for them^32^. However, in the latter case the synthesis is performed by using fungal cell-free extracts^33^. One of the most popular and frequently used NPs are AuNPs. Synthesis of AuNPs can be grouped into four main specific categories depending on the processing and the exploitation of the fungal mycelia. In the most basic approach, washed mycelia can be used directly to produce the NPs intracellularly (i.e. *in vivo*) as discussed above, although in this case the extraction of the NPs from the mycelia before further application is necessary^31,34–36^. With the *in vitro* production of NPs this step can be eliminated. Three possible *in vitro* methods are reviewed in the following section. Firstly, the supernatant of the fermented fungi, containing extracellular proteins and other secreted compounds can be used for biosynthesis of AuNPs as well^25,37^. The drawback of this method is that some remaining components of the fermentation media are present during the reaction. In other approaches, the intracellular components of the fungi are capable of producing biologically synthesized gold nanoparticles (BioAuNPs) after the disruption of the cells^38,39^. Lastly, the aqueous extract in which the mycelia was kept suspended can be used for the biosynthesis as well^27,40,41^. Processes occurring during the incubation are most likely autolysis of the cells followed by dissolution of membrane proteins and surface carbohydrates into the aqueous solution. This method has a drawback as well, since the washing and resuspending of the mycelia in sterile conditions are challenging tasks, therefore bacterial contaminations can lead to false results. On the other hand this can be overcome by the use of antibiotics, but these can influence and facilitate the production of NPs^42^. It should be noted that the use of antibiotics is not environmentally friendly. Therefore, their usage in the large scale production of BioAuNPs should be avoided.

In most publications in the literature one or two out of the four methods mentioned above have been used for synthesis and no comprehensive study is available on the comparison between the different approaches.

**Figure 1:**
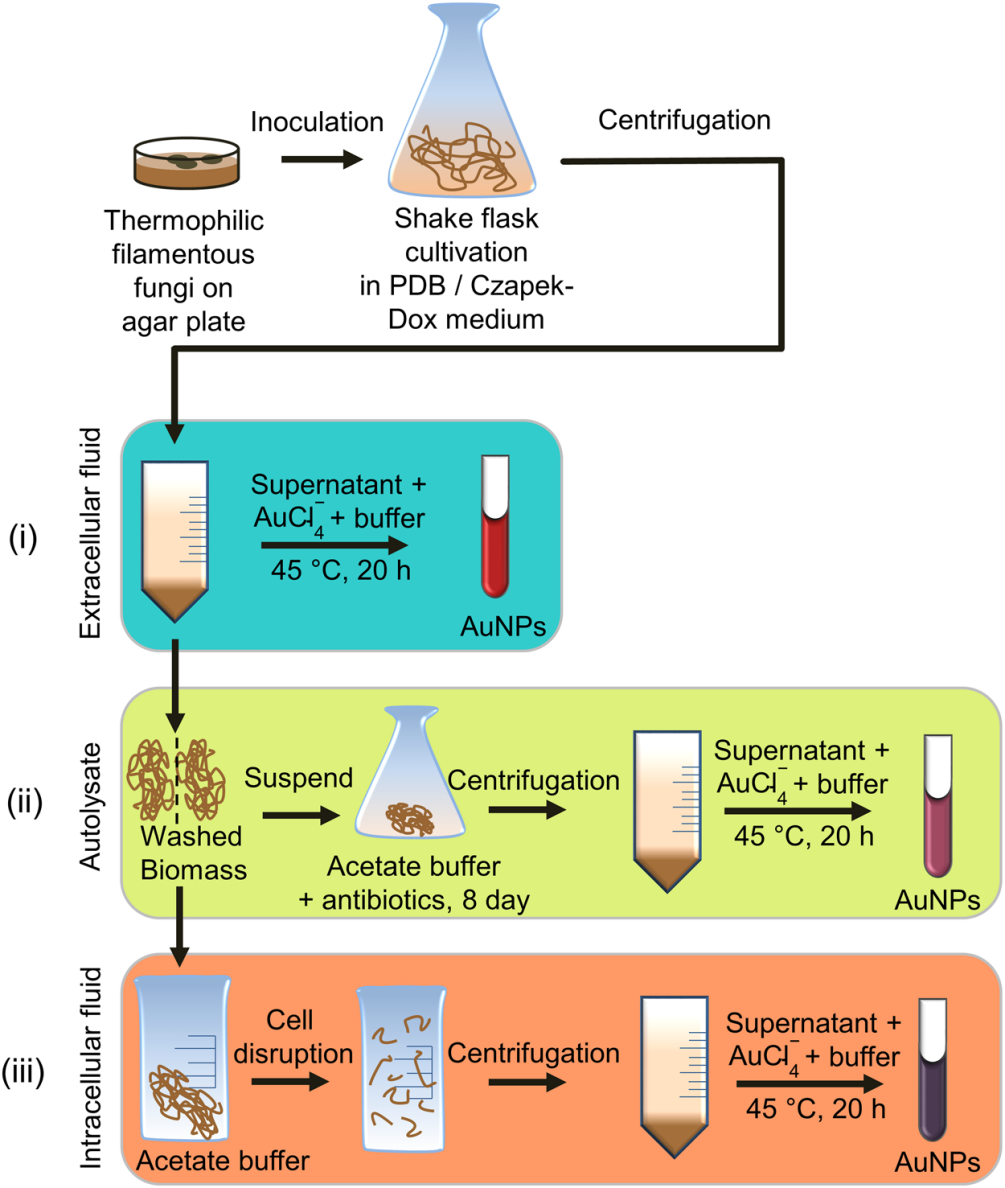
The sketch of the preparation of different extracts from fungi and synthesis of AuNPs by different methods using (**i**) extracellular extract, (**ii**) autolysate and (**iii**) intracellular extract.

In this study we used 29 thermophilic filamentous fungal strains for the synthesis of AuNPs from a gold salt. One of our main goals was to give an overall picture about how the above-mentioned methods are connected to each other and how the different down-stream processes influence the quality and characteristics of the produced BioAuNPs. We investigated the effect of the supernatant of the fermented fungi (extracellular fraction), the autolysate of the fungi and the cell-free intracellular extract of the mechanically disrupted cells on the characteristics and quality of AuNPs (Fig. 1). We omitted the application of pure living mycelia from the study, because our main goal was to elaborate a simple one-step green synthesis method to produce AuNPs for further modifications and applications. The intracellular production of BioAuNPs requires further extraction and purification steps making the synthesis more complicated.

## Results and Discussion

### Effect of growth media on nanoparticle production under thermophilic conditions – control experiments

We carried out different control experiments under thermophilic conditions to investigate and clarify the effect of the growth media of fungi on the production of AuNPs. These are key experiments to distinguish between the effect of the growth media and fungi-originated compounds regarding the synthesis of AuNPs, which, to our best knowledge, has been unknown in the literature. Synthesis of NPs consists of two simultaneous processes, reduction of Au (III) into Au(0) producing nuclei followed by growth of particles (nucleation and growth) and stabilization of produced NPs by capping ligands. These protecting ligands prevent the further growth and coagulation of particles due to electrostatic or steric stabilization. Sometimes one chemical compound can act as a reducing as well as a capping agent (for examples citrates or amino acids).

**Figure 2:**
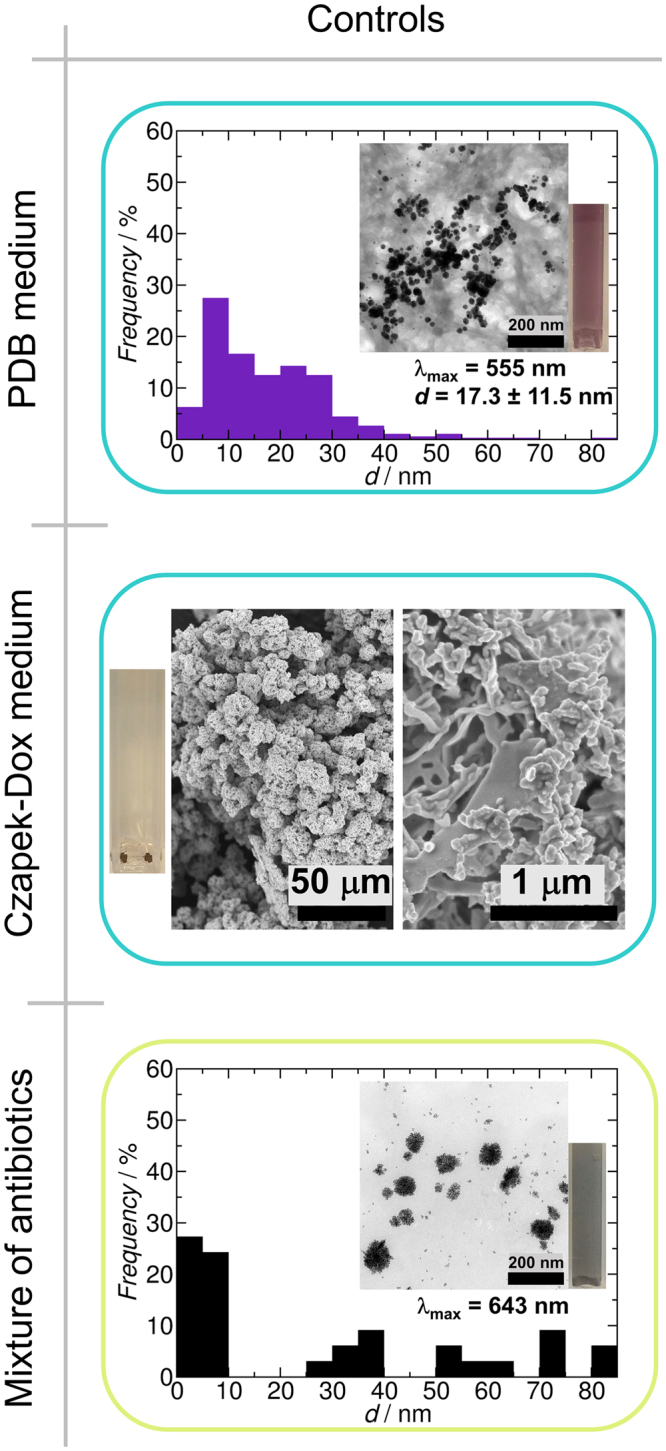
Effect of different growth media on the synthesis of AuNPs (control experiments). PDB medium reduces gold salt and provide AuNPs with an average size of 17.3 nm with standard deviation of 11.5 nm (top panel- size distribution of produced NPs with TEM micrograph and a cuvette containing the solution of AuNPs). Modified Czapek-Dox medium produces no NPs, but microscopic gold precipitate (middle panel- SEM micrographs and a cuvette), which sediments from the solution. An antibiotic mixture of ampicillin and kanamycin can produce highly polydisperse AuNPs (bottom panel - size distribution of produced NPs with TEM micrograph and a cuvette containing the solution of AuNPs).

Potato-dextrose broth (PDB) is a complex medium, containing biomolecules ranging from small molecules such as sugars and amino acids to polymers like potato-starch. Some of these compounds can reduce gold salts and partially stabilize produced AuNPs in water phase^43–47^. In our experiments PDB control (sterile, uninoculated medium) provided stable BioAuNPs with a characteristic purple colour and a wavelength of maximum absorption peak in the UV-Vis spectra at 555 nm (Fig. 2). TEM analysis showed that the BioAuNPs produced by the PDB control were varied in size ranging from 1 to 80 nm and had mostly spherical and hexagonal shape.

BioAuNP production of the PDB itself justifies the application of a synthetic, defined medium, such as the modified Czapek-Dox medium, which is also an often used medium for fermentation of fungi producing AuNPs, even though it is not an optimal medium for growing numerous strains^34,36,48^. The modified Czapek-Dox medium contains glucose as a defined carbon source, which by itself is a reducing agent and can reduce Au(III) to Au(0)^49^. The reaction with modified Czapek-Dox control (sterile, uninoculated medium) resulted in no NPs only black precipitate (Fig. 2), which is reasonable, because there was no capping, but only a reducing agent present in the solution. These influencing and contributing factors should be considered when BioAuNPs are produced with the secreted proteins and other biomolecules. Additionally, before processing the mycelia, the mycelial biomass should be carefully washed in order to remove all remnants of the growth media.

In our protocol, when we used intracellular cell-free protein extract, mycelia were washed three times with an acetate buffer solution, and after this process the supernatant provided negative results for production of NPs (we observed no colour change in the solution and found no NPs in TEM micrographs).

In order to obtain aqueous autolysate solution, either sterile conditions should be maintained, or some antibiotics need to be used to avoid bacterial contamination. Unfortunately, antibiotics can also behave like reducing and somewhat capping agents, as it has been shown in the literature^42^ and our experiments as well (Fig. 2). Mixture of ampicillin and kanamycin was used against broad variety of bacteria, and the control (reaction of gold salt with the solution of ampicillin and kanamycin) provided a positive result and resulted in a grey BioAuNP solution. The wavelength of the maximum absorption peak in the UV-VIS spectra was at 643 nm, TEM analysis showed that the particles were very polydisperse, and the sample contained mostly aggregates of amorphously shaped particles (Fig. 2).

### Screening for BioAuNP production by thermophilic fungi grown on potato-dextrose broth (PDB)

**Figure 3:**
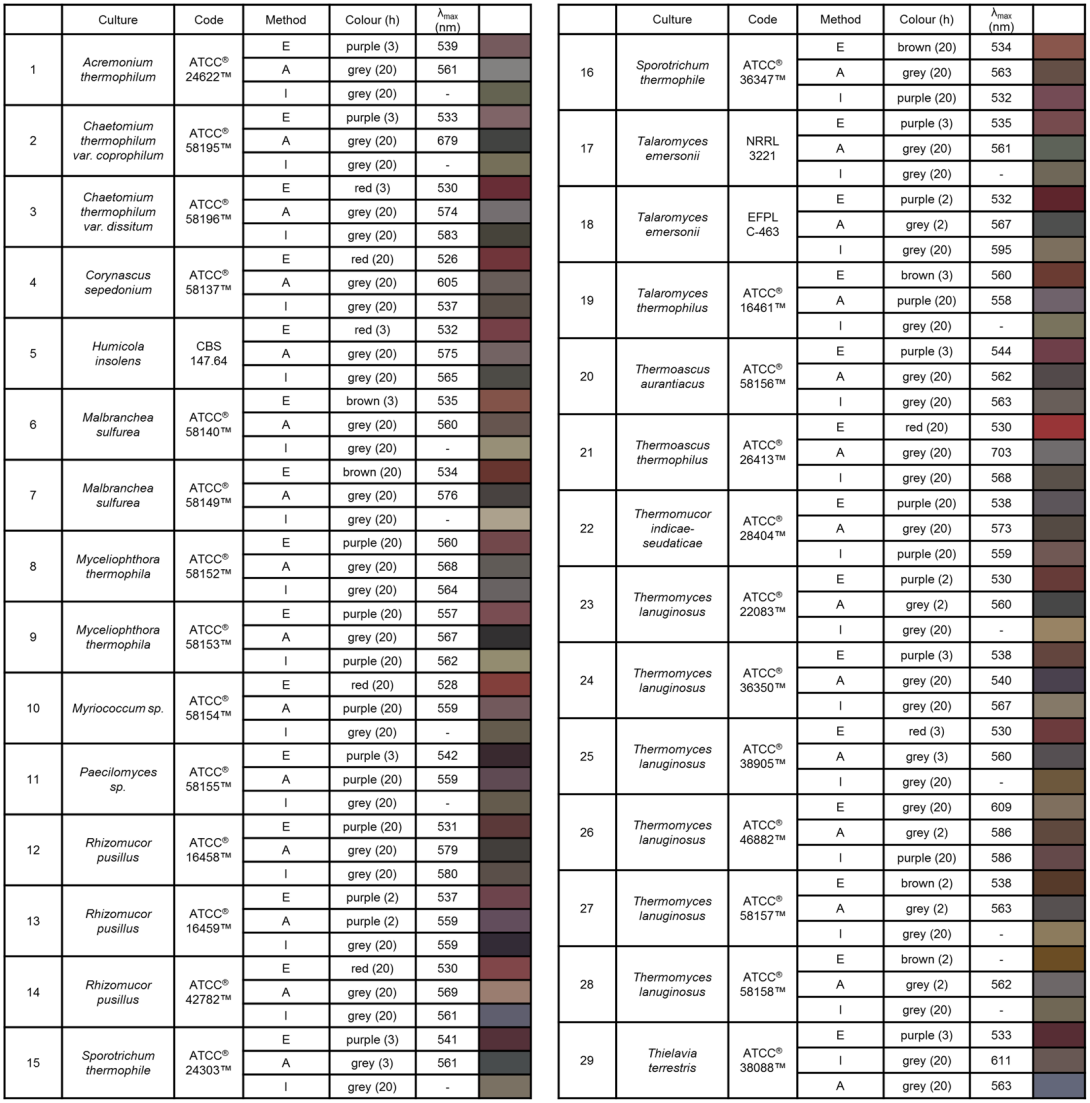
Characterization of BioAuNPs synthetized by thermophilic fungi grown on PDB medium: E: extracellular fraction; A: autolysate fraction, I: intracellular fraction. Numbers in the brackets correspond to the elapsed time in hours after the colour (shown in the last column) of the sample did not change further.

For the comprehensive screening, we propagated the fungi on PDB, as it is a complex medium ideal for testing different strains, and it has been widely used for the synthesis of AuNPs^27,31^. The fermented mycelia were processed with three different methods (Fig. 1) for 29 thermophilic fungi and the samples were tested for the production of BioAuNP. The pre-trials were conducted at room temperature (25.0 ± 0.3 °C), 35.0 ± 0.3 °C, 45.0 ± 0.3 °C, 55.0 °C and 80.0 ± 0.3 °C in order to test the effect of temperature on the characteristics of NPs. The resulting spectra (colours) did not show significant differences for the different temperatures, only the reaction rate of NPs formation was higher with the increasing temperature (data not shown). Further experiments (synthesis of NPs) were carried out at 45.0 °C as the fermentation temperature was the same. The colours of the samples were recorded in the first, second and third and 20th hours from the start of the experiments. The absorbances of the samples were measured by UV-Vis spectrophotometry after 20 hours for all 29 strains processed with the three different down-stream methods. The summarized results are shown in Fig. 3. The fermentations, sample preparations and reactions were repeated several times (at least three times) to ensure reproducibility. Thermophilic fungi grown on PDB medium can produce a wide variety of AuNPs, differing in colour from red through purple to grey and ranging in the wavelength of the maximum absorption peak in the UV-Vis spectra from around 530 to 600 nm indicating formation of various sized particles with different size distributions. It should be noted that the supernatant of the fermented mycelia – containing the remnants of the growth medium and new proteins and metabolites produced the BioAuNPs – produced NP solutions with lower maximum absorption peaks compared to the samples using intracellular and autolysate fluids. The lower corresponding wavelengths indicate smaller and more monodisperse particles. Intracellular and autolysate samples resulted mostly in greyish colours and higher absorption peaks (if there was any peak at all, see Fig. 3).

### Comparison of PDB and modified Czapek-Dox media for the synthesis of BioAuNPs by thermophilic fungi

After the screening of the thermophilic fungal strains on PDB medium, several strains were chosen for testing the formation of BioAuNPs on a synthetic medium. Synthetic (defined) medium such as modified Czapek-Dox with glucose as carbon source has the advantage of minimizing the disturbing factors (e.g., reduction potential of its components) in comparison with multi-component PDB medium.

**Figure 4:**
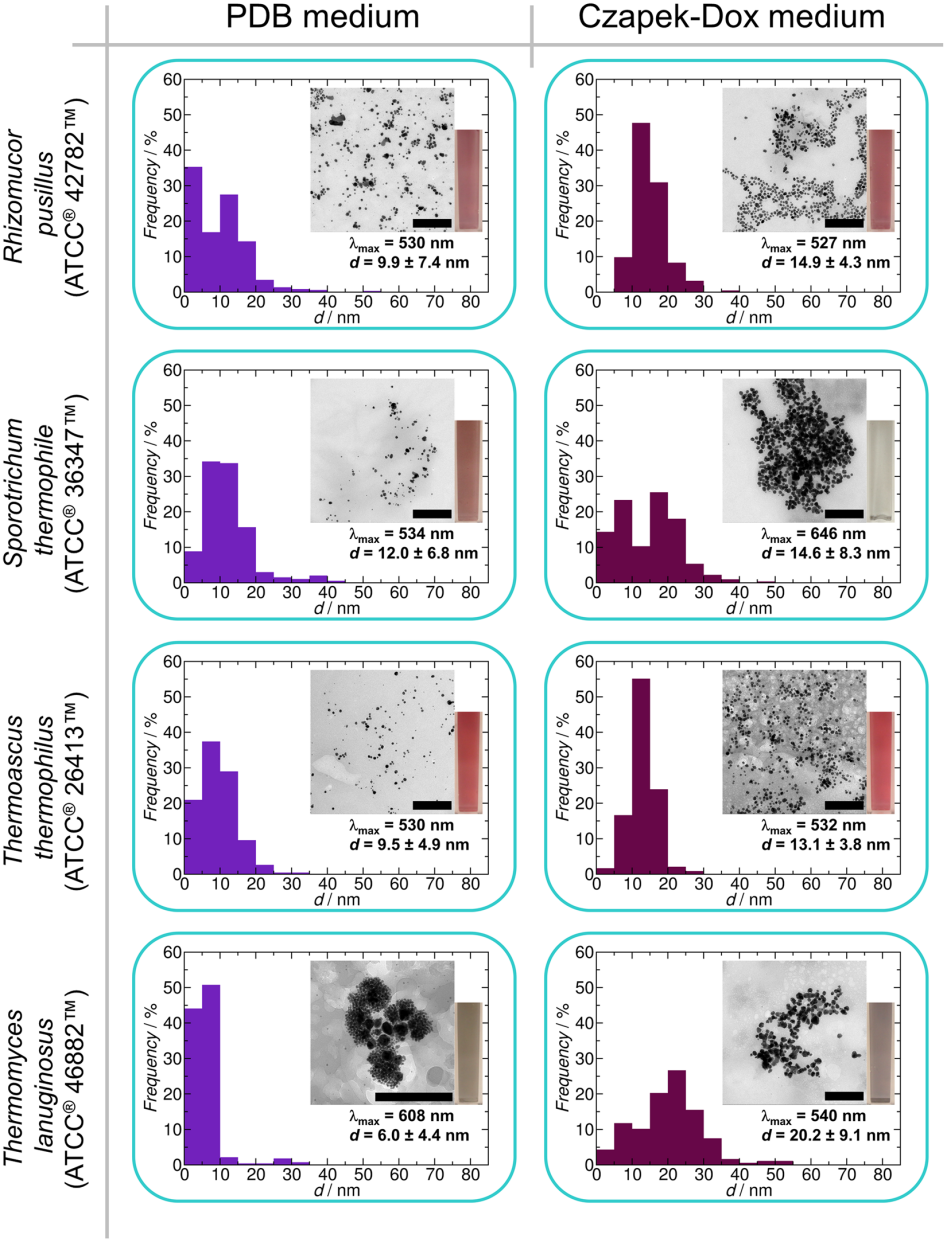
Synthesis of AuNPs using extracellular extract of various fungi (*Rhizomucor pusillus* ATCC^®^ 42782™, *Sporotrichum thermophile* ATCC^®^ 36347™, *Thermoascus thermophilus* ATCC^®^ 26413™ and *Thermomyces lanuginosus* ATCC^®^ 46882™). Each graph shows the size distribution of produced BioAuNPs with TEM micrograph (inset), the average size with standard deviation of the particles and the wavelength of maximum absorbance and a photo of a cuvette containing solution of AuNPs. The scale bar represents 200 nm.

**Figure 5:**
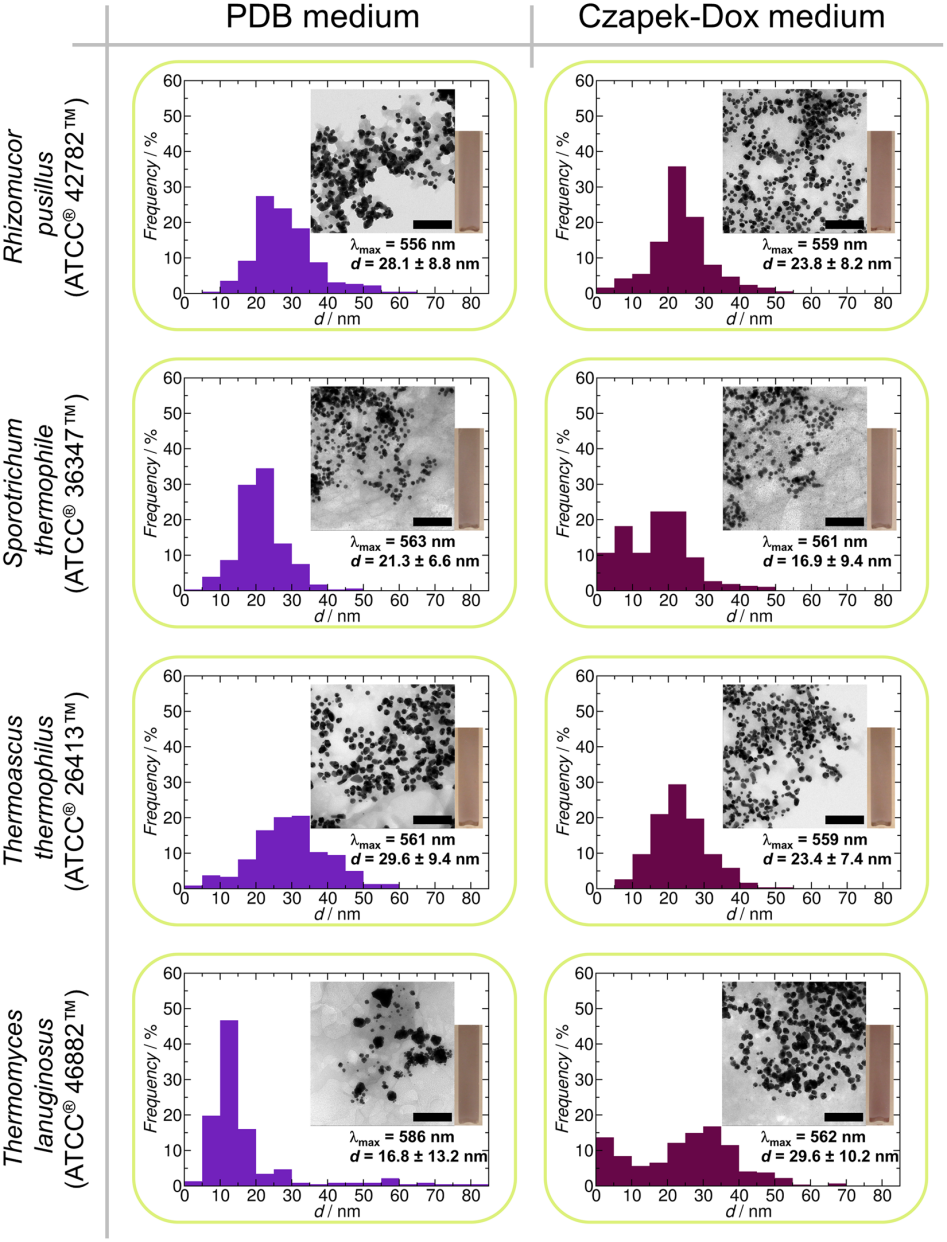
Synthesis of AuNPs using autolysate of various fungi (*Rhizomucor pusillus* ATCC^®^ 42782™, *Sporotrichum thermophile* ATCC^®^ 36347™, *Thermoascus thermophilus* ATCC^®^ 26413™ and *Thermomyces lanuginosus* ATCC^®^ 46882™). Each graph shows the size distribution of produced BioAuNPs with TEM micrograph (inset), the average size with standard deviation of the particles, the wavelength of maximum absorbance and a photo of a cuvette containing solution of AuNPs. The scale bar represents 200 nm.

**Figure 6:**
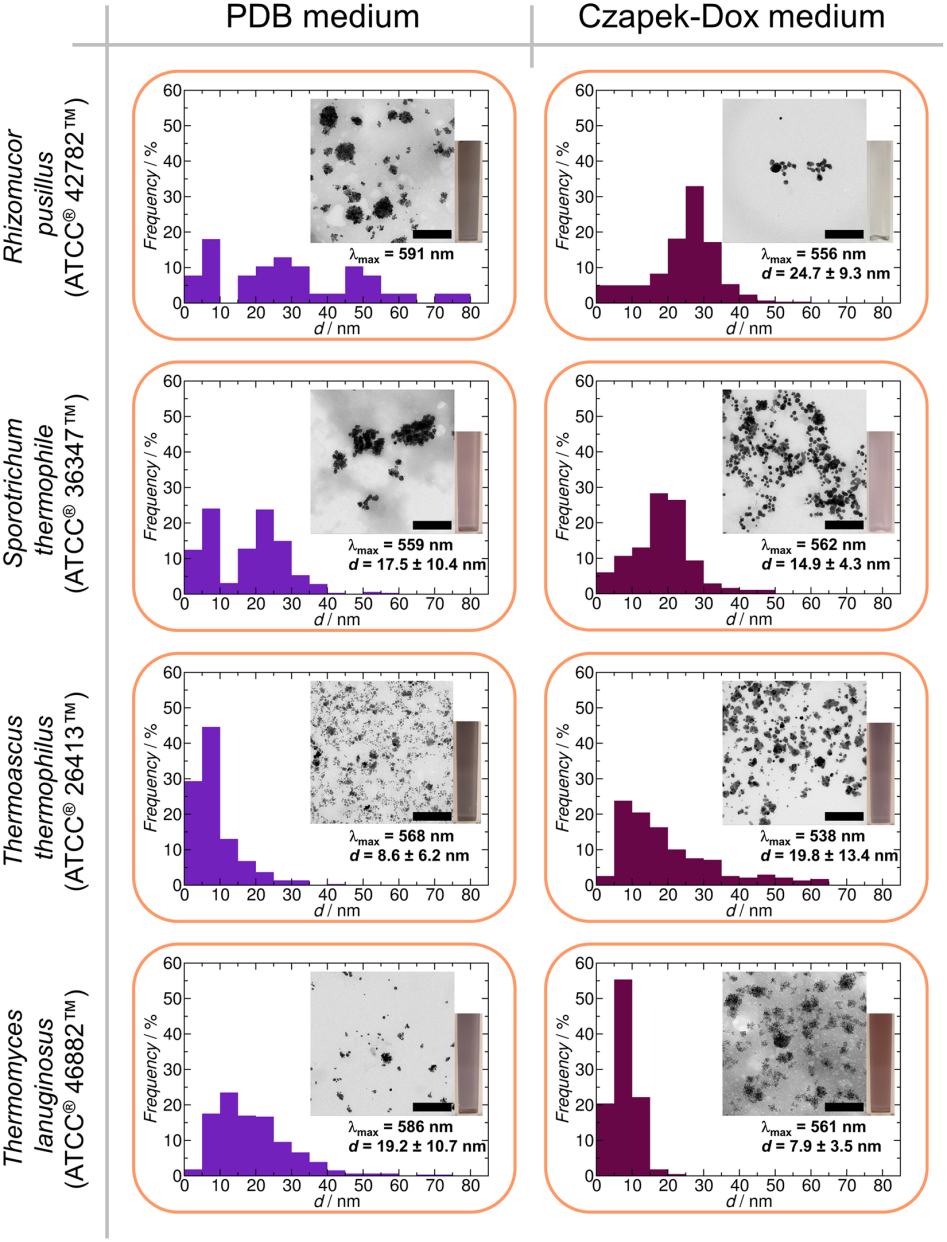
Synthesis of AuNPs using the intracellular extract of various fungi (*Rhizomucor pusillus* ATCC^®^ 42782™, *Sporotrichum thermophile* ATCC^®^ 36347™, *Thermoascus thermophilus* ATCC^®^ 26413™ and *Thermomyces lanuginosus* ATCC^®^ 46882™). Each graph shows the size distribution of produced BioAuNPs with TEM micrograph (inset), the average size with standard deviation of the particles, the wavelength of maximum absorbance and a photo of a cuvette containing solution of AuNPs. The scale bar represents 200 nm.

In the end, four strains (*Rhizomucor pusillus* ATCC^®^ 42782™, *Sporotrichum thermophile* ATCC^®^ 36347™, *Thermoascus thermophilus* ATCC^®^ 26413™ and *Thermomyces lanuginosus* ATCC^®^ 46882™) were chosen to compare the effects of the two culturing media and the processing methods on the synthesis of AuNPs. The BioAuNPs were characterized with UV-Vis spectrophotometry and TEM. The results can be seen in Figs 4–6. There are two main conclusions that can be drawn from these investigations. First, there are no overall correlations between effect of the growth media on the characteristics of the synthesized AuNPs (size and size distribution). Secondly, however, the applied down-stream methods affect only slightly the quality of the produced BioAuNPs. Extracellular extract provides AuNPs with an average size of 6–12 nm and 13–20 nm using PDB and modified Czapek-Dox media, respectively. Autolysate generates bigger particles with an average size of between 17 and 30 nm irrespectively of the growth media. The polydispersity of the autolysate-generated samples is the smallest among the three down-stream methods (39% in average). Intracellular extract produces the most polydisperse samples (54% in average), and the average size of the particles ranges between 8 and 40 nm.

### Synthesis of BioAuNPs by fractions of extracellular liquid (supernatant) separated by using ultracentrifugal filtration

Recently, several studies published have shown that biomolecules such as amino acids^43,44^, peptides^50^, proteins^51,52^, mono- and polysaccharides^45,46,49,53^, cofactors^54,55^, melanin^56^ and others^18,20^ can be used for synthesizing AuNPs. In order to identify the responsible biomolecules for the formation of BioAuNPs by thermophilic fungi, the separation of the biomolecules by molecular size was carried out with PALL Microsep™ Advance Centrifugal Devices for the selected supernatants of the extracellular fluid. We tested the extracellular fluid of *Thermoascus thermophilus* ATCC^®^ 26413™ cultured on a modified Czapek-Dox medium since the fermentation process proved to be reliable and the produced BioAuNPs were homogeneous in size and shape.

**Figure 7:**
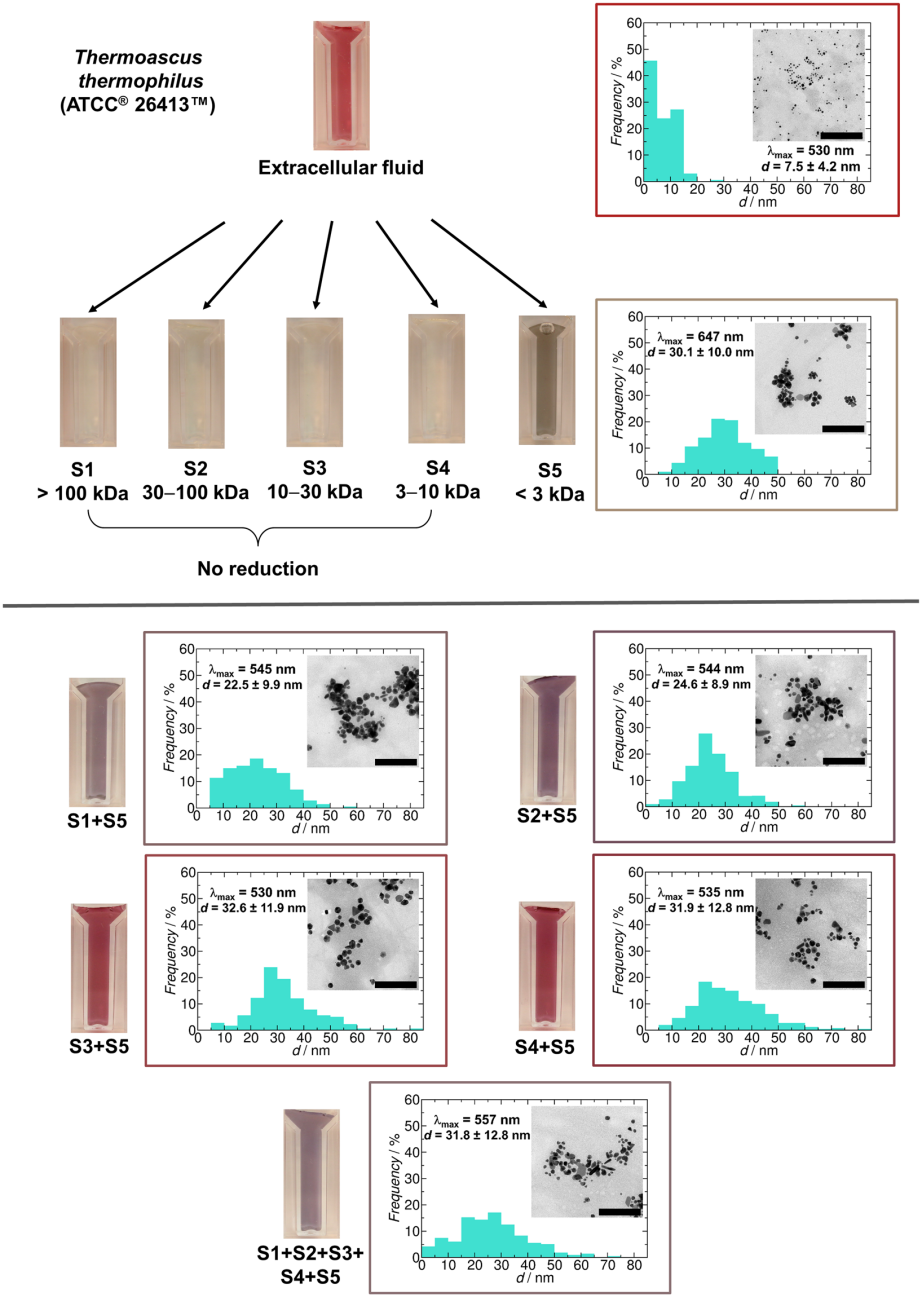
Separation of the biomolecules by molecular weight in the extracellular fluid of *Thermoascus thermophilus* ATCC^®^ 26413™ cultured on a modified Czapek-Dox medium by using molecular sieves and the effect of fractions on the formation of AuNPs (colour, morphology, the average size with standard deviation and size distribution of the particles). The scale bar represents 200 nm.

The results are summarized in Fig. 7. Sample fractions S1, S2, S3 and S4 contain biomolecules with high molecular weights, most likely proteins and other biopolymers. Fraction S5 is a more complex sample, containing all small molecular weight biomolecules, including peptides, mono- and oligosaccharides, primary metabolites, amino acids, cofactors and many others. Biomolecules over 3 kDa secreted by this fungal strain are not capable of reducing Au(III) to Au(0), and we observed no formation of AuNPs. However, biomolecules under 3 kDa reduce Au(III) to Au(0) and produce AuNPs. The average size of the particles (~30 nm) is greater than in case of using all extracellular fractions (~ 8 nm) and the sample is more polydisperse. We observed that the synthesized NPs were not stable in water phase and sedimented over 20 hours. It is likely because the fraction can reduce Au(III) and facilitate NPs formation, but small components from this fraction cannot sufficiently stabilize NPs in the water phase because of small molecular chain.

When mixing fractions S1, S2, S3 and S4 with S5, we found that all reactions provided different colours from red to purple with different absorption peaks indicating AuNP formation in all cases. The samples were more stable compared to the sample produced by fraction S5. TEM analysis of particles revealed that the average size of particles shifted towards higher values (~30 nm) with higher polydispersity of the particles compared to a native sample.

These results confirm that there are two steps of the production of BioAuNPs by thermophilic fungi, the reduction of Au(III) to Au(0) followed by the stabilization of the core of NPs by capping agents. The reducing agents can be very diverse chemical species such as monosaccharides^45,49^, antibiotics^42^, amino acids^43,44^, cofactors^54,55^ or other molecules like melanin^56^. The capping agents should be biopolymers larger than 3 kDa (most likely protein), and our results imply that there is no specific protein responsible for the stabilization of NPs since all fractions above 3 kDa were able to stabilize the solution of BioAuNPs. Additionally, the amino acid residues of the proteins can bind non-specifically to the surface of the NPs, providing additional stabilization. These findings are in accordance with other publications^37,40^. Interestingly, none of the mixed samples could provide similar characteristics of AuNPs to the original extracellular sample. This indicates that the synthesis of BioAuNP is a more complicated process than a few proteins and reducing factors interacting with each other.

## Conclusion

In this study we investigated the formation of AuNPs using 29 thermophilic fungal strains fermented on two growth media (PDB and modified Czapex-Dox). The shake flask fermentation material was processed by three different methods: (1) centrifugation of broth in order to obtain supernatant liquid; (2) preparing washed mycelia for autolysis and using the autolysate liquid; and (3) mechanical cell disruption of the properly washed mycelia and use of debris-free liquid for synthesis of AuNPs. We observed formation of NPs practically in all cases when we used the three processing methods of mycelia grown on PDB. We obtained similar positive results in case of using a synthetic medium (modified Czapek-Dox).

In the green synthesis of NPs, a widely used method is the application of the extracellular extracts produced by the centrifugation of the whole fermentation broth. We pointed out that in this case the supernatant inevitably contains the remnants of the growth media that can reduce a gold salt and/or stabilize the formed NPs. In case of PDB, these components can be glucose, amino acids and proteins, each of them capable of reducing Au(III) to Au(0). The use of synthetic medium can decrease the number of reducing agents, however, it contains glucose as carbon source, and it cannot be guaranteed that all glucose would be consumed by fungi during their fermentation process. Additionally, even if the growth medium contains sucrose (which is a nonreducing sugar), at elevated temperature its hydrolysis provides glucose and fructose, therefore it can contribute to the formation of AuNPs^57^. This highlights an important aspect that in this biotechnological method, when extracellular extracts are used for synthesis of NPs, the effect of fungi-originated compounds on the formation of NP cannot be separated from the effect and contribution of the remnants of the growth media. To show the clear effect of fungi-originated compounds on the formation of AuNPs, the fermented mycelia should be washed and processed further. In this case the supernatant of the washed mycelia provided negative results for formation of NPs. Therefore, for example cell disruption provides liquid which contains chemical species from the inner part of cell and cell wall region of the fungi (primary and secondary metabolites, enzymes, DNA and RNA fragments, etc.) characteristic to the given isolate. Using this intracellular extract, we were able to find and provide a direct evidence of the effect of the fungi-originated chemical species on the production of AuNPs. Additionally, we could evaluate the size of the possible reducing and capping agents using ultracentrifugal filtration. Based on this investigation we can draw conclusion that the size of reducing agents is less than 3 kDa, and these chemical species can be glucose, amino acids and cofactors. These chemical compounds can efficiently reduce Au(III), but cannot stabilize NPs. Most probably the capping agents are bigger biomolecules than 3 kDa (e.g., protein), and these can stabilize the formed AuNPs. Proteins can be bonded onto the surface of AuNPs through the Van der Waals attraction between the protein and the surface of NPs or dative bonding between the metal and the conducting electrons of nitrogen and sulphur atoms of the proteins. Proteins can be strongly attached onto the metal surface close to their isoelectric point, away from this pH this bonding ability decreases^58–60^. The main conclusion of our study is that the fungi-originated chemical compounds can be used for synthesis of AuNPs, however, one should be very careful when the mycelia would be processed further, because the effect of the growth media can contribute to the formation of NPs.

## Materials and Methods

### Chemicals

Chloroauric acid (HAuCl_4_·3H_2_O), and the other pure chemicals and biochemicals were purchased from Sigma-Aldrich, potato dextrose broth was obtained from Lab M (Bury, United Kingdom).

### Microorganisms, maintenance and pre-inoculum preparations

The thermophilic filamentous fungi were received from leading microbial culture collections such as ATCC (American Type Culture Collection), CBS (Centraalbureau voor Schimmelcultures), NRRL (Northern Regional Research Center) and EFPL (Eastern Forest Products Laboratory). Vegetative growth and sporulation were performed on potato dextrose agar (PDA) medium at 45 °C for 7 days in Petri dishes.

### Biological synthesis of gold nanoparticles with secreted biomolecules

Filamentous fungi were grown in potato dextrose broth (PDB) containing glucose 20.0 g/L or modified Czapek-Dox media containing glucose 20.0 g/L; NaNO_3_ 3.0 g/L; K_2_HPO_4_ 1.0 g/L; MgSO_4_·7H_2_O 0.5 g/L; KCl 0.5 g/L; FeSO_4_·7H_2_O 0.01 g/L in distilled water and the pH was set to 7.3. Shake flask fermentations were performed in 500 ml Erlenmeyer flasks with 100 ml working volume at 45 °C and 200 rpm on a rotary shaker for 3 days in the case of PDB medium, and for 4–10 days for modified Czapek-Dox media. The culture was centrifuged at 15000 relative centrifugal force (RCF) at 4 °C for 20 min (Sigma Laboratory Centrifuges 6K15, 12169-H rotor), the supernatant was used to examine the secreted proteins, and the harvested cells were washed three times with an acetate buffer solution (pH = 4.7) to remove any residual media components before further work. In a typical experiment, 100 µL of 0.01 M tetrachloroauric acid trihydrate was mixed in test tubes with 450 µL of fungal cell-free extract and 450 µL acetate buffer (pH = 4.7), and it was set at 35.0 °C, 45.0 °C and 55.0 °C for 3–20 hours.

### Biological synthesis of gold nanoparticles with autolysate

For the autolysate fraction (including cell-wall and cell-membrane attached and intracellular biomolecules), approximately 4 g of washed wet mycelia were suspensed in 30 mL acetate buffer (pH = 4.7), completed with 100 µg/ml ampicillin and 50 µg/ml kanamycin, for 8 days at 24 °C and then the aqueous solution was separated by centrifugation (4 °C, 15000 RCF, 20 min). The supernatant was used for the reactions, as carried out for the secreted and intracellular biomolecules.

### Biological synthesis of gold nanoparticles with intracellular biomolecules

For the intracellular fraction, approximately 4 g of washed wet mycelia was homogenized with Ultra-Turrax® TP 18–10 for 7 minutes in 30 mL acetate buffer (pH = 4.7). Then the cell free protein extract was separated by centrifugation (4 °C, 15000 RCF, 20 min). The supernatant was used for the reactions, as carried out for the secreted biomolecules.

### Separation of biomolecule fractions in the extracellular liquid by ultracentrifugal filtration

PALL Microsep™ Advance Centrifugal Devices with the molecular weight cut-off values of 3 kDa, 10 kDa, 30 kDa and 100 kDa were used to separate the biomolecules of the extracellular fluid into five distinct fractions: S1, biomolecules larger than 100 kDa; S2, biomolecules between 30 and 100 kDa; S3, biomolecules between 10 and 30 kDa; S4, biomolecules between 3 and 10 kDa; S5, biomolecules smaller than 3 kDa. Sigma Laboratory Centrifuges 6K15 was used at 7500 RCF for 30 min for each step. After each separating step, the permeate was transferred to the next Microsep™ tube with lower molecular weight cut-off value, and the retentate was washed three times and then resuspensed in acetate buffer (pH = 4.7), except for fraction S5, which is the last permeate itself. The samples were cooled during the centrifugation process to avoid the degradation of the biomolecules. The fractions were used for the reactions, as described earlier.

### Characterization of AuNPs

The UV-Vis spectrum of the AuNP solutions were recorded in the range of 450–650 nm (Varian CARY 3 UV-Vis spectrophotometer). The particle size and morphology of the gold nanoparticles were investigated by transmission electron microscopy (FEI TECNAI G^2^ 20 × -Twin high-resolution transmission electron microscope). The solution was applied onto carbon-coated copper grids without any further dilution or purification.
